# Prognostic Implications of Chronic Heart Failure and Utility of NT-proBNP Levels in Heart Failure Patients with SARS-CoV-2 Infection

**DOI:** 10.3390/jcm10020323

**Published:** 2021-01-17

**Authors:** Laia C. Belarte-Tornero, Sandra Valdivielso-Moré, Miren Vicente Elcano, Eduard Solé-González, Sonia Ruíz-Bustillo, Alicia Calvo-Fernández, Isaac Subinara, Paula Cabero, Cristina Soler, Héctor Cubero-Gallego, Beatriz Vaquerizo, Núria Farré

**Affiliations:** 1 Heart Failure Unit, Department of Cardiology, Hospital del Mar, 08003 Barcelona, Spain; svaldivielso@psmar.cat (S.V.-M.); esolegonzalez@parcdesalutmar.cat (E.S.-G.); sruiz@psmar.cat (S.R.-B.); 61725@parcdesalutmar.cat (A.C.-F.); 2 Heart Diseases Biomedical Research Group (GREC), IMIM (Hospital del Mar Medical Research Institute), 08003 Barcelona, Spain; pcabero@psmar.cat (P.C.); csoler@psmar.cat (C.S.); hcuberogallego@psmar.cat (H.C.-G.); bvaquerizo@psmar.cat (B.V.); 3 Department of Cardiology, Hospital del Mar, 08003 Barcelona, Spain; mvicenteelcano@psmar.cat; 4 Department of Medicine, School of Medicine, Universidad Autonoma de Barcelona, 08003 Barcelona, Spain; 5 CIBER Epidemiology and Public Health, IMIM-Parc de Salut Mar, 08003 Barcelona, Spain; isubirana@imim.es

**Keywords:** COVID-19, SARS-CoV-2, coronavirus, heart failure, prognosis, biomarkers, NT-proBNP, troponin

## Abstract

Background: The prevalence and prognostic value of chronic heart failure (CHF) in the setting of Severe Acute Respiratory Syndrome Coronavirus 2 (SARS-CoV-2) infection has seldom been studied. The aim of this study was to analyze the prevalence and prognosis of CHF in this setting. Methods: This single-center study included 829 consecutive patients with SARS-CoV-2 infection from February to April 2020. Patients with a previous history of CHF were matched 1:2 for age and sex. We analyze the prognostic value of pre-existing CHF. Prognostic implications of N terminal pro brain natriuretic peptide (NT-proBNP) levels on admission in the CHF cohort were explored. Results: A total of 129 patients (43 CHF and 86 non-CHF) where finally included. All-cause mortality was higher in CHF patients compared to non-CHF patients (51.2% vs. 29.1%, *p* = 0.014). CHF was independently associated with 30-day mortality (hazard ratio (HR) 2.3, confidence interval (CI) 95%: 1.26–2.4). Patients with CHF and high-sensitivity troponin T < 14 ng/L showed excellent prognosis. An NT-proBNP level > 2598 pg/mL on admission was associated with higher 30-day mortality in patients with CHF. Conclusions: All-cause mortality in CHF patients hospitalized due to SARS-CoV-2 infection was 51.2%. CHF was independently associated with all-cause mortality (HR 2.3, CI 95% 1.26–4.2). NT-proBNP levels could be used for stratification risk purposes to guide medical decisions if larger studies confirm this finding.

## 1. Introduction

The SARS-CoV-2 pandemic has caused a high number of hospitalizations and mortality worldwide. Patients with pre-existing cardiovascular (CV) disease [1,2] show a worse prognosis than those without pre-existing CV diseases; with a reported mortality 5 to 10 times higher [3].

SARS-CoV-2 infection has been associated with direct viral injury of cardiomyocytes, microvascular dysfunction, small vessels thrombotic complications and systemic inflammation; all of which could cause cardiac injury and precipitate an acute cardiovascular syndrome [4] (i.e., acute heart failure, myocarditis, pericarditis, vasculitis, cardiac arrhythmias and cardiac arrest) [5].

Heart failure (HF) decompensation is one of the main causes of hospitalization worldwide and is associated with high in-hospital mortality [6,7]. Respiratory infections trigger up to 10% of total HF hospital admissions, being the most common non-cardiovascular cause for hospitalization [8]. In addition, respiratory viral and bacterial infections worsen prognosis of HF patients [9]. Notably, previous SARS-CoV and Middle East Respiratory Syndrome (MERS) coronavirus epidemics have also been associated with acute heart failure [10].

The age and CV comorbidities are associated with poor prognosis in SARS-Cov-2 infection. There is scarce data about the prognosis of coronavirus 2019 (COVID-19) disease in patients with pre-existing HF. New date is emerging about the impact of chronic heart failure (CHF) in patients with COVID-19 [11,12]. Recent studies have shown that mortality could reach 40% in patients with CHF [11]. Some of these studies did not analyze CHF as a separate entity; and others only included patients with advanced HF [13,14,15]. However, it remains unclear if CHF, in an elderly population with high comorbidity, is associated per se with a higher mortality [16]. Therefore, the aim of this study was to analyze the prevalence and prognosis of CHF in a cohort of patients with SARS-Cov-2 infection.

## 2. Materials and Methods

### 2.1. Study Design and Data Collection

This single-center study consecutively enrolled all patients admitted to hospital with symptoms and signs related with COVID-19 and positive nasopharyngeal swab-polymerase chain reaction (PCR) for SARS-CoV-2 infection from February to April 2020. There were no exclusion criteria. All clinical procedures and treatments were performed according to local protocol for SARS-CoV-2 infection. The primary endpoint was to determine whether a history of CHF was an independent factor for 30-days mortality among patients hospitalized with SARS-CoV-2 infection. As secondary endpoints, we analyzed the clinical factors associated with 30-day mortality as well as the potential prognostic value of high-sensitivity T troponin (Hs-TnT) and N terminal pro brain natriuretic peptide (NT-proBNP) in patients with CHF. 

Clinical variables related to medical background, COVID-19 symptoms and hemodynamic and respiratory status on admission were recorded. The medical charts of patients with a history of CHF were reviewed. Those who fulfilled the HF definition by the European Society of Cardiology Guidelines were included [17]. Development of acute decompensated heart failure (AHF) was defined as new signs and symptoms of HF during admission that responded to diuretics and vasodilators and with evidence of functional or structural heart abnormality [17]. In CHF patients, it was collected HF ethology, baseline New York Heart Association (NYHA) functional class and left ventricle ejection fraction (LVEF). Blood samples were collected within the first 48 h after admission, including the cardiac biomarkers Hs-TnT and NT-proBNP. Chest X-ray and pharmacological and non-pharmacological treatment as well as the need for respiratory support were registered. Death and cause of death were collected.

### 2.2. Statistical Analysis

**Figure 1:**
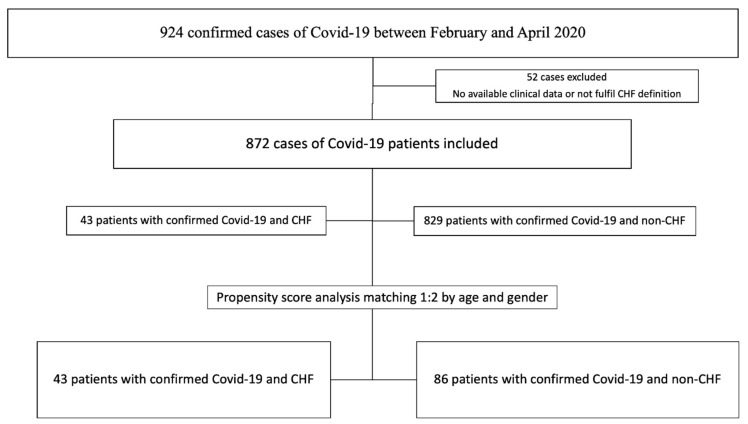
Flow chart of patient’s selection and propensity score matching. CHF: chronic heart failure.

Baseline characteristics between the patients with and without HF in the whole cohort are summarized in the Supplemental Table S1. Because the non-CHF cohort was remarkably different from the CHF patients by being almost 20 years younger and having very low prevalence of CV comorbidities, we did a propensity score analysis matching 1:2 according to age (±years) and sex. As shown in Table 1, after matching by age and sex, major prognostic factors previously related with 30-day mortality in COVID19 infection were balanced in both groups. The flowchart of the study can be found in Figure 1. Categorical variables are presented as percentage and compared with Chi-squared or Fisher test. Continuous variables are presented as mean and standard deviation (SD) or median and 25 and 75 percentiles for variables with non-normal distribution. Continuous variables are compared with the Student *t*-test or non-parametric tests according to its distribution.

The univariate and adjusted hazard ratio (HR) of death for CHF were analyzed using Cox proportional hazard models. The models were adjusted for potential confounders selected by stepwise backward inclusion, among patient characteristics that were significantly (*p*-value < 0.05) associated with baseline CHF as well as with the endpoint. Kaplan–Meier survival estimates were used to calculate the 30-day observed cumulative incidence of death, and statistical significance was tested by the log-rank test.

For biomarkers analysis only patients with Hs-TnT or NT-proBNP determination on admission were included. In the CHF cohort a receiver operation characteristic (ROC) analysis was done to select the Hs-TnT and NT-proBNP cut-off values better related to 30-day mortality in terms of sensibility and specificity. Cumulative survival curves of 30-days mortality were estimated using Kaplan–Meier analysis for the NT-proBNP cut-off value identified in the ROC analysis.

*p*-values < 0.05 were considered statistically significant. Propensity score matching was done with R Statistical Package version 3.6.1 (R: A language and environment for statistical computing; R Foundation for Statistical Computing, Vienna, Austria) and all other tests were performed with SPSS version 25 (IBM Corporation, Armonk, NY, USA).

### 2.3. Ethics Considerations

The study was performed in accordance with the provisions of the Declaration of Helsinki, ISO 14155 and clinical practice guidelines. The study protocol was approved by the Institutional Ethics Committee and the hospital’s research commission (number CEIm 2020/9178). Oral informed consent was obtained but the need for written informed consent was waived in light of the infectious disease hazard.

## 3. Results

### 3.1. Characteristics of Chronic Heart Failure (CHF) versus Non-CHF

From February to April 2020, 43 patients (4.9%) with previous medical history of CHF were hospitalized with SARS-CoV-2 infection. After propensity score matching 129 patients were included for analysis (43 with previous CHF; and 86 without previous CHF) (Figure 1). Baseline characteristics of both groups are described in Table 1. Briefly, mean age in both groups was 80.3 (±12) years and more than 80% patients had at least one CV risk factor. Despite propensity score matching, hypertension, obesity, atrial fibrillation, valvular heart disease, asthma and chronic kidney disease were more frequent in CHF patients.

Clinical presentation of SARS-CoV-2 infection included fever in 50% of patients in both groups. The most common symptom in CHF failure patients compared with non-CHF patients was dyspnea with or without cough (62.8% vs. 43%; *p* < 0.038). Up to 80% of patients in both groups presented with bilateral pulmonary infiltrates suggestive of viral pneumonia. Other clinical and laboratory findings on admission are described in Table 2. CHF patients had lower hemoglobin (11.7 vs. 12.8 g/dL, *p* < 0.008) and estimated glomerular filtration rate (eGFR) (53 vs. 67 mL/min/1.73 m^2^, *p* < 0.008) on admission. Hs-TnT and NT-proBNP levels on admission were recorded in 103 and 81 patients respectively. More than 75% of patients in both groups had basal abnormal Hs-TnT levels. Both Hs-TnT and NT-proBNP levels on admission were significantly higher in CHF patients (41.6 (21.4–69) vs. 22.8 (14–34.2) ng/L, *p* < 0.003, and 3423 vs. 558 pg/mL, *p* < 0.002, respectively). In-hospital treatments are described in Table 2. Need for any respiratory support, including oxygen supplementation, high flow nasal cannula, non-invasive or invasive ventilation, was similar in both groups. Most patients (73.6%) only received oxygen support during admission.

### 3.2. Outcomes in CHF vs. Non-CHF

The median hospital stay was 7 days longer in CHF patients (17 vs. 10 days, *p* < 0.023). Overall mortality was remarkably higher in CHF patients compared to non-CHF patients (51.2% vs. 29.1%, *p* = 0.014) as shown in Table 1. Median time from SARS-CoV-2 infection diagnosis to death was shorter in CHF patients (14 (5–25) days vs. 20 (10–36) days; *p* < 0.006). Respiratory failure was the main cause of death in both groups (89.8% of total deaths) and CV death was more frequent in CHF patients (9.3% vs. 0%, *p* < 0.019). All CV deaths were due to decompensated HF refractory to treatment. In addition, 21% of CHF patients developed an AHF decompensation during admission, compared to only 3.5% of non-CHF patients (*p* < 0.004) (Table 1).

**Figure 2:**
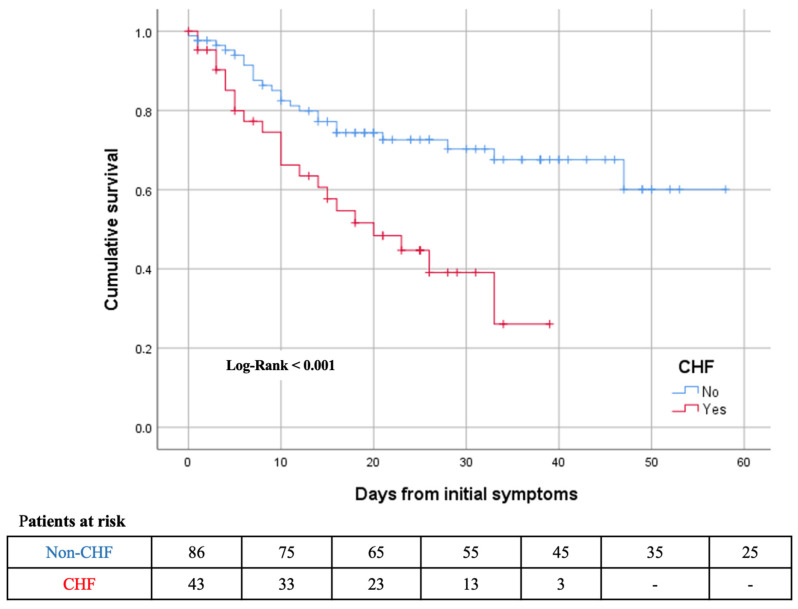
Kaplan–Meier 30-days survival curves for mortality by previous chronic heart failure (CHF) during time from initial symptoms.

Baseline characteristics of patients who died compared to those who survived are summarized in Table 3. Patients who died were older, had a higher prevalence of CHF, diabetes mellitus, ischemic heart disease, AF, peripheral vascular disease and CKD. Hs-TnT and NT-proBNP levels on admission were higher in patients who died. After adjusting by age and comorbidities, CHF remained as an independent risk factor for 30-day mortality (adjusted HR: 2.3; 95% CI: 1.25–4.2; *p* < 0.014). Patients’ hazard ratios of 30-day mortality predictors and the Kaplan–Meier survival curves for previous CHF are presented in Table 4 and Figure 2, respectively.

### 3.3. CHF Cohort

Mean LVEF was 53.7% (±14.3) in CHF patients and 69.8% had a preserved ejection fraction, defined as a LVEF ≥50%. Only 20.9% of patients had a LVEF ≤ 40%. Most patients (76.7%) were stable in NYHA ≤ 2 class previous to SARS-CoV-2 infection with a low proportion of patients with advanced HF (18.6%). Detailed information about CHF patients is showed in Table 5.

**Figure 3:**
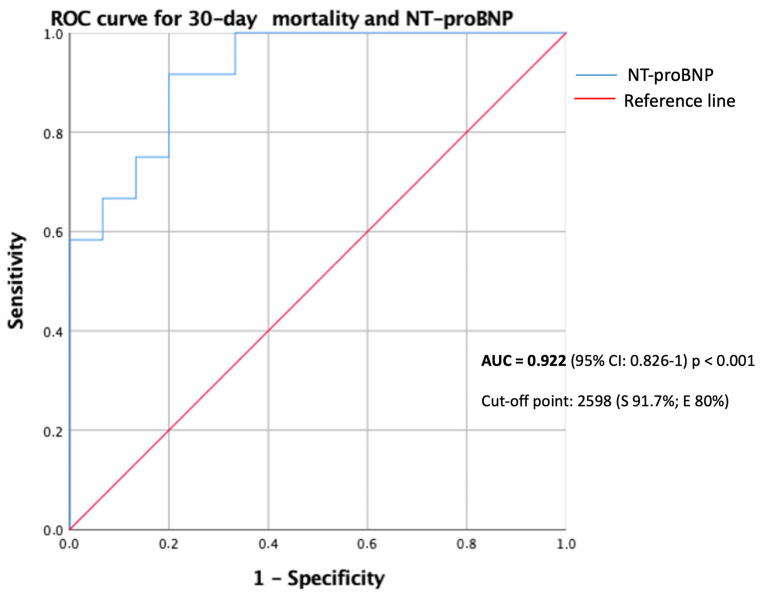
ROC (receiver operating characteristic) curve for 30-day mortality and NT-proBNP.

**Figure 4:**
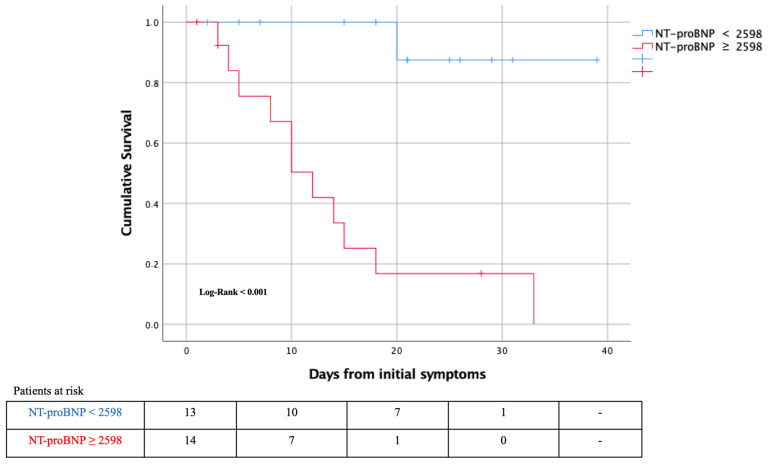
Kaplan–Meier 30-day survival curve for mortality by NT-proBNP during time from initial symptoms.

Half of the patients (22 of 43; 51.2%) with CHF died during the study period (Table 1). Predictors of death in patients with CHF are described in Table 6. The only baseline characteristics associated with worse prognosis in this population were age, LVEF ≤ 40%, atrial/flutter and chronic kidney disease. Background HF medical therapy was not related with mortality in this study. Hs-TnT on admission was measured in 74.4% of patients (*n* = 32). Almost 85% of patients had Hs-TnT values above normal range on admission. According to ROC curves the best cut-off of Hs-TnT in CHF patients for predicting 30-day mortality was 38.27 ng/L (AUC = 0.798 (95%CI:0.645–0.95), *p* < 0.004, sensitivity 78.6%, specificity 66.7%). However, a normal HsTnT on admission was associated with a 100% survival (HR 0.48, 95%CI: 0.33–0.71). NT-proBNP on admission was measured in 63% (*n* = 27) of patients and mean value was 3423 (616–10,400) pg/mL. According to ROC curves (Figure 3) the best cut-off of NT-proBNP in CHF patients for predicting 30-days mortality was 2598 pg/mL (sensitivity 91.7%, specificity 80%). Patients with CHF and NT-proBNP above 2598 pg/mL on admission had higher risk of death (78.6% vs. 7.7%; *p* < 0.001; HR 10.2, 95%CI: 1.5–68.5) (Figure 4).

## 4. Discussion

Several aspects of this study are worth highlighting. In the present study, 5% of patients with COVID-19 had pre-existing CHF. This was an elderly population with a high comorbidity. During hospital stay, 21% of CHF had an AHF decompensation and half of the patients died. An NT-proBNP > 2598 pg/mL had an excellent sensitivity in predicting mortality in patients with previous diagnosis of HF.

Notably, given the epidemiological relevance of CHF [16], few studies have analyzed the prevalence and prognosis of CHF in patients with SARS-CoV-2 infection [11,12]. Prevalence of CHF in the setting of SARS-CoV-2 infection has been described to be between 4.1% to 36.5%, these differences might be due to the different populations analyzed [11,12,13,14,18]. Although this prevalence seems low, it is possible that CHF patients were especially careful in self-isolating due to their baseline high risk; thus, their risk of infection could be lower than in the general population. More than 65% of patients had heart failure with preserved ejection fraction (HFpEF), probably because HFpEF is more prevalent in the general population, especially in older patients [19]. Moreover, these results are in line with previous studies of COVID in CHF patients, even though other cohorts included a much younger population [11]. 

As expected, patients with CHF were older and had more CV comorbidities compared to non-CHF patient. Comorbidities, CV risk factors and older age have been associated with poor prognosis during COVID-19 disease [1,20,21,22]. Thus, it may be difficult to assess the role CHF per se played in mortality. To minimize this limitation, first we did a propensity score matching for age and gender. Second, we carried out a survival analysis with Cox proportional hazard models. Our results showed that 30-day mortality in CHF patients was remarkably high, almost double in comparison with non-CHF patients (51.2% vs. 29.1%). Similar mortality rate (40 to 63%) has been described in other series [11,12,13,14], which reflects the extremely poor prognosis in this population. The presence of CHF was independently associated with all-cause death in our cohort (HR 2.3 CI95% (1.26–4.2), *p* = 0.007), confirming similar results recently published by Álvarez et al. [11]. Interestingly, several treatments have been studied in COVID [23,24,25,26]. At the time this cohort was recruited, several anti-viral treatments, hydroxychloroquine plus azithromycin and tocilizumab were used, which have now been abandoned due to lack of efficacy. Moreover, although anticoagulation seems to play a role in COVID treatment [27,28], its use was not standardized and the protocols describing the indications and optimal doses varied throughout the study. We did not see a difference in prognosis according to the treatment received.

Viral infections are common causes of HF exacerbations [29]. Acute HF decompensation associated to SARS-CoV-2 infection can occur as the first clinical manifestation of the infection even in patients without previous CV disease [30]. Moreover, AHF developed during SARS-CoV-2 infection has been associated with an increased risk of mortality [12,31]. In the present study, around 4% of patients without previous history of HF had an acute episode of HF during hospitalization; this could be caused by myocardial involvement of virus infection in a cohort of elderly patients with high CV comorbidity [32]. Similar findings were described in a study by Rey et al. regarding the prevalence of AHF in patients with COVID-19 disease [12]. AHF was remarkably more frequent in patients with previous CHF affecting almost 1 out of 4 CHF patients (21 vs. 3.5%, *p* < 0.019). Notably, CHF patients who develop an acute HF decompensation during hospitalization for COVID19 disease had an in-hospital mortality of 44%. One challenge in identifying HF decompensation is that clinical manifestations, as well as radiological findings, can be difficult to distinguish from respiratory infection. That requires a high degree of suspicion and the use of special image technics as computed tomography or lung ultrasound [33] in order to promptly initiate HF medication to optimize loading conditions, especially when there is associated respiratory or hemodynamic compromise. In our cohort all CV deaths were due to HF decompensation refractory to treatment.

There are several factors that could explain this high mortality other than the older age and the presence of comorbidities. First, previous studies have demonstrated that HF confers a proinflammatory status to patients that may weaken the immunological response to virus infections [34]. Second, SARS-CoV-2 infection has been associated with markedly elevated proinflammatory mediators and cytokine profile similar to the cytokine release syndrome [4]. Moreover, SARS-CoV-2 infection has been associated with direct myocardial injury that might worsen previous cardiac diseases such as CHF [3,35] and prevent achieving the higher hemodynamic demands associated with infection [4,36]. Finally, SARS-CoV-2 infection has been associated with angiotensin-converting enzyme 2 (ACE2) signaling pathways [36]. Patients with CHF have an upregulated renin-angiotensin-aldosterone system [34]. Binding ACE2 may have special impact in these patients and explains the deleterious impact on survival.

The usefulness of cardiac troponin and NT-proBNP as prognostic markers in CHF is well recognized [37]. Similarly, biochemical markers of myocardial injury have been associated with higher mortality in SARS-CoV-2 infection suggesting their potential role as a risk stratification tool [18,32,38,39]. There is scarce data about the prognostic value of these biomarkers in CHF patients with COVID-19. Dong et al. has described severe myocardial injury in patients with end-stage HF during COVID-19 and its association with disease progression and mortality [15]. In our HF cohort, 84% of patients had Hs-TnT measurement above normal values, probably due to preceding myocardial injury and more susceptible myocardium to virus insult. Although troponin values should be interpreted with caution in CHF population due to chronic myocardial injury [18], it seems to be a relationship between Hs-TnT on admission and prognosis. In fact, Hs-TnT levels below normal range were associated with better prognosis in the present study. Considering that the Hs-TnT cut-off value identified by ROC curves was rather low and associated with low sensitivity and specificity, we consider that the easiest approach would be to consider any patients with Hs-TnT above > 14 ng/L as a high-risk patient. The majority of previous studies focused on troponin release as the biomarker associated with acute myocardial injury in non-selected populations [4,18]. A recent study that focused on CHF patients admitted to hospital with SARS-CoV2 infection shows an association between increased troponin concentrations and in-hospital mortality in this specific population. However, NT-proBNP was not significantly associated with mortality [11]. The prognostic value of NT-proBNP in viral respiratory infections has been previously described [40]. A recent study conducted in a non-selected population of patients admitted to hospital with SARS-CoV2 infection underlined that a NT-proBNP level > 300 pg/mL on admission was an independent predictor of mortality or need for mechanical ventilation. The authors also emphasize that the NT-proBNP even improved the prognostic accuracy of Hs-TnT for the outcomes analyzed [32]. In patients with severe COVID-19, an NT-proBNP > 88.64 pg/mL on admission was independently associated with in-hospital mortality suggesting its usefulness as a specific index of COVID19 disease severity [41]. The overall median NT-proBNP described in the present study was high in both groups probably due to old age and high prevalence of CV risk factors and disease in our population. As expected, NT-proBNP levels were higher in CHF patients. However, a cut-off of NT-ProBNP > 2598 ng/dL on admission was strongly associated with poor prognosis in CHF patients infected with SARS-CoV-2.

The present clinical study has certain limitations. This is a single center study with a relatively small sample size; therefore, these results would benefit from a validation cohort. Biomarkers results should be interpreted with caution because we only focused on a single measurement on admission without being systematically collected in all patients. Finally, given the difficulty in establishing a HF diagnosis in the setting of acute respiratory failure and pulmonary infiltrates, it is possible that episodes of acute HF were not identified.

## 5. Conclusions

Patients with CHF admitted due to SARS-CoV-2 have a 51.2% all-cause mortality rate and this higher risk was maintained after multivariate analysis (HR 2.3, CI 95% 1.26–4.2). Acute HF decompensation was frequent (21%) and all CV deaths were due to HF refractory to treatment. If larger studies confirm these results, a combination of these biomarkers might be used to establish initial risk stratification on admission and guide clinical decisions in this high-risk population.

## Figures and Tables

**Table 1:** Comparison of baseline characteristics and outcomes between patients with and without chronic heart failure (CHF).

	Total (*n* = 129)	CHF (*n* = 43)	Non-CHF (*n* = 86)	*p* Value
Women	66 (51.2)	22 (51.2)	44 (51.2)	1
Age, years	80.3 (±12)	80.3 (±12.1)	80.4 (±12.1)	0.984
Influenza vaccination	27 (34.6)	10 (43.5)	17 (30.9)	0.287
Smoking	31 (24)	9 (20.9)	22 (25.6)	0.832
Diabetes	49 (38)	21 (48.8)	28 (32.6)	0.073
Hypertension	101 (78.3)	38 (88.4)	63 (73.3)	0.050
Dyslipemia	67 (51.9)	27 (62.8)	40 (46.5)	0.081
Obesity	29 (26.4)	14 (32.6)	15 (17.4)	0.018
Ischemic cardiac disease	22 (17.1)	10 (23.3)	12 (14)	0.185
AF or flutter	31 (24)	22 (51.2)	9 (10.5)	0.001
Valvular heart disease	19 (14.7)	14 (32.6)	5 (5.8)	0.001
ACEI or ARB II	58 (45)	23 (53.5)	35 (40.7)	0.169
COPD	14 (10.9)	8 (18.6)	6 (7.0)	0.069
Previous Cancer	33 (25.6)	10 (23.3)	23 (26.7)	0.669
CKD	34 (26.4)	18 (41.9)	16 (18.6)	0.005
Peripheral vascular disease	17 (13.2)	7 (16.3)	10 (11.6)	0.462
Stroke	21 (16.3)	9 (20.9)	12 (14.0)	0.312
Asthma	14 (10.9)	9 (20.9)	5 (5.8)	0.015
LVEF, %	57 (±11.9)	54 (±12.6)	63 (±6.9)	0.002
Cardiac Biomarkers *				
Hs-TnT > 14 ng/L	80 (77.6)	27 (84.4)	53 (74.6)	0.273
Hs-TnT, ng/L	25.4 (14.5–47.4)	41.6 (21.4–69)	22.8 (14–34.2)	0.003
NT-proBNP, pg/mL	841 (228–3785)	3423 (616–10,400)	558 (213–1692)	0.002
**Outcomes**				
Intensive care unit admission	7 (5.4)	1 (2.3)	6 (7.0)	0.423
Advanced ventilatory support	16 (12.4)	4 (9.3)	12 (14.0)	0.450
Hospital length of stay, days	12 (3–24)	17 (8–31)	10 (1–20)	0.023
Clinical worsening during admission	39 (30.2)	14 (32.6)	25 (29)	0.749
Overall death	47 (36.4)	22 (51.2)	25 (29.1)	0.014
Overall CV death	4 (3.1)	4 (9.3)	0 (0)	0.019
Acute HF during admission	12 (9.3)	9 (21)	3 (3.5)	0.004

Results are expressed as mean and (standard deviation), or number and (percentage). ACEI: Angiotensin-converting-enzyme inhibitors, ARB: Angiotensin II receptor blockers. CHF: Chronic Heart failure, COPD: chronic obstruction pulmonary disease, CKD: chronic kidney disease, AF: atrial fibrillation, CV: cardiovascular. HF: Heart failure. NT-proBNP, N-terminal probrain natriuretic peptide. Hs-TnT: high-sensitivity troponin-T. LVEF: Left ventricular ejection fraction. * only for patients with biomarkers on admission.

**Table 2:** Clinical presentation, laboratory findings on admission and in-hospital treatment of patients stratified by heart failure.

	Total (*n* = 129)	CHF (*n* = 43)	Non-HF (*n* = 86)	*p* Value
**Clinical findings**				
Fever	62 (48)	23 (53.5)	39 (45.3)	0.214
Dyspnea with or without cough	64 (49.6)	27 (62.8)	37 (43)	0.038
SBP, mmHg	133 (±21.4)	133 (±22.7)	133 (±20.9)	0.995
DBP, mmHg	74 (±15.2)	73 (±15.7)	74 (±15.1)	0.786
Heart rate, bpm	88 (±16.7)	89 (±20)	89.5 (±19.9)	0.564
Respiratory rate, rpm	27 (±6.8)	27 (±7.5)	27 (±6.5)	0.902
Oxygen saturation, %	94 (±5.7)	94 (±6.8)	94 (±5)	0.331
Initial FiO2, %	31 (±23.7)	32 (±25)	30 (±23.2)	0.671
PaO2/FiO2	271 (±127.6)	312 (±135.4)	256 (±121.9)	0.080
PaO2/FiO2 < 300	50 (38.8)	13 (30.2)	37 (43)	0.387
Laboratory findings *				
Hemoglobin, g/dL	12.5 (±2.0)	11.7 (±2.3)	12.8 (±1.8)	0.008
WBCC, per µL	8.3 (±5.0)	9.3 (±6.4)	7.8 (±4.1)	0.177
Lymphocytes, per µL	1.3 (±1.6)	1.5 (±2.4)	1.2 (±1)	0.299
Platelet count	215 (±99)	233 (±131)	206 (±80)	0.243
Creatinine, mg/dL	1.3 (±0.9)	1.6 (±1.2)	1.2 (±0.6)	0.024
eGFR, mL/min/1.73m2	62 (±28)	53 (±25.8)	67 (±28.4)	0.008
AST, U/L	41 (±58)	39 (±53)	42 (±59)	0.870
ALT, U/L	35 (±61)	39 (±53)	41 (±65)	0.611
Bilirubin, mg/dL	1.1 (±6.3)	2.7 (±11.7)	0.4 (±0.17)	0.293
CPK, U/L	87 (51–194)	59 (41.5–167.5)	98 (58.8–209)	0.144
Serum lactate, mmol/L	2.2 (1.1–1.8)	1.4 (1.1–2.1)	1.5 (1.1–1.7)	0.840
Baseline CRP, mg/dL	9.6 (5.2–17)	8.8 (3.9–12.7)	10.6 (5.3–19.5)	0.122
Procalcitonin, ng/mL	0.18 (0.1–0.4)	0.2 (0.1–0.5)	0.17 (0.1–0.4)	0.675
LDH, U/L	345 (±154)	339 (±130)	348 (±163)	0.811
D-dimer, ng/mL	876 (402–1440)	880 (560–1995)	835 (575–1535)	0.936
Abnormal chest radiography	109 (84.5)	35 (81.4)	74 (86)	1
**In-hospital treatment**				
Lopinavir/Ritonavir	13 (10.1)	3 (7)	10 (11.6)	0.542
Darunavir/Ritonavir	2 (1.6)	0 (0)	13 (15.1)	0.552
Corticosteroid	47 (36.4)	13 (30.2)	34 (39.5)	0.294
Tocilizumab	13 (10.1)	3 (7)	10 (11.6)	0.542
Hydroxychloroquine ± Azithromycin	114 (88.4)	35 (81.4)	79 (91.9)	0.080
Enoxaparin (prophylaxis or treatment doses)	74 (57.4)	19 (44.2)	55 (64)	0.136
Vitamin D	25 (19.4)	6 (14)	19 (22.1)	0.270
Ceftriaxone	79 (61.2)	21 (48.8)	58 (67.4)	0.041
**Respiratory support**				
Oxygen support	95 (73.6)	35 (81.4)	60 (69.8)	0.436
High Flow Nasal Cannula	1 (0.8)	0 (0)	1 (1.2)	
Non-invasive ventilation	8 (6.2)	3 (7)	5 (5.8)	
Intubation and invasive ventilation	7 (5.4)	1 (2.3)	6 (7)	

Results are expressed as mean and (standard deviation), or median and (interquartile range), or number and (percentage). SBP: Systolic blood pressure. DBP: Diastolic blood pressure. WBCC: White blood cell count. AST: Aspartate transaminase. ALT: Alkaline transaminase. CPK: Creatine phosphokinase. LDH: Lactate dehydrogenase. CRP: C-reactive protein, eGFR: Estimated glomerular filtration rate. * on admission.

**Table 3:** Comparison of baseline characteristics between patients alive and dead during hospitalization.

	Alive (*n* = 82)	Dead (*n* = 47)	*p* Value
Previous CHF	21 (25.6)	22 (46.8)	0.014
Women	37 (45.1)	29 (61.7)	0.070
Age, years	77.4 (±13.7)	85.5 (±5.5)	0.001
Smoking	20 (24.4)	11 (23.4)	0.508
Diabetes	21 (25.6)	28 (59.6)	0.001
Hypertension	60 (73.2)	41 (87.2)	0.062
Dyslipemia	40 (48.8)	27 (57.4)	0.343
Obesity	16 (19.5)	13 (27.7)	0.175
Ischemic cardiac disease	9 (11.0)	13 (27.7)	0.015
AF or flutter	15 (18.3)	16 (34)	0.044
Valvular heart disease	10 (12.2)	9 (19.1)	0.283
ACEI or ARA II	37 (45.1)	21 (44.7)	0.961
COPD	8 (9.8)	6 (12.8)	0.597
Previous Cancer	21 (25.6)	12 (25.5)	0.992
Stroke	14 (17.1)	7 (14.9)	0.747
Asthma	11 (13.4)	3 (6.4)	0.217
CKD	13 (15.9)	21 (44.7)	0.001
Peripheral vascular disease	7 (8.5)	10 (21.3)	0.040
Influenza vaccination	16 (19.5)	11 (23.4)	0.313
Hs-TnT *, ng/L	20.5 (14–30.5)	44 (26–78)	0.001
NT-proBNP *, pg/mL	482 (180–893)	3786 (1391–10,400)	0.001
LVEF, %	59.5 (±8.8)	53.7 (±14.7)	0.44

Results are expressed as mean and (standard deviation), or number and (percentage). ACEI: Angiotensin-converting-enzyme inhibitors, ARB: Angiotensin II receptor blockers. CHF: Chronic Heart failure, COPD: chronic obstruction pulmonary disease, CKD: chronic kidney disease, AF: atrial fibrillation, CV: cardiovascular. NT-proBNP, N-terminal probrain natriuretic peptide. Hs-TnT: high-sensitivity troponin-T. * only for patients with biomarkers on admission.

**Table 4:** Hazard ratios of 30-day death for previous CHF adjusted for potential confounders.

	Univariate HR (95%CI)	*p* Value	Adjusted * HR (95%CI)	*p* Value
Previous CHF	1.76 (1.13–2.73)	0.014	2.3 (1.26–4.2)	0.007
Age (per every year)	1.1 (1.03–1.12)	0.002	1.08 (1.03–1.14)	0.001
Diabetes	4.41 (1.52–3.82)	0.001	3.04 (1.65–5.6)	0.001
Peripheral vascular disease	1.78 (1.11–2.87)	0.040	2.49 (1.17–5.3)	0.018
Ischemic cardiac disease	1.86 (1.19–2.9)	0.015	-	
AF or flutter	1.63 (1.04–2.55)	0.044	-	
CKD	2.26 (1.48–3.44)	0.001	-	

* Model adjusted for age, previous CHF, diabetes, peripheral vascular disease, ischemic cardiac disease, AF or flutter, CKD. HR: Hazard ratios. CI: Confidence interval. CHF: Chronic heart failure. AF: Atrial fibrillation. CKD: Chronic kidney disease.

**Table 5:** Clinical characteristics of CHF patients.

**LVEF**	
Preserved	30 (69.8)
Mid-range	2 (4.7)
Reduced	9 (20.9)
**NYHA**	
I	12 (27.9)
II	21 (48.8)
III	8 (18.6)
**HF etiology**	
Ischemic	5 (11.6)
Non-ischemic	18 (41.9)
Hypertensive	17 (39.5)
**Background medical therapy**	
RAAS inhibition	23 (53.5)
Beta-blockers	14 (32.6)
ARNI	4 (9.3)
MRA	4 (9.3)
Loop diuretics	17 (39.5)
Thiazides	3 (7)
Anticoagulant	14 (32.6)
Antiplatelet	9 (20.9)
Statins	15 (34.9)

Results are expressed as number and (percentage). LVEF: Left ventricle ejection fraction. NYHA: New York Heart Association Functional Class. HF: Heart Failure. RAAS: renin-angiotensin-aldosterone system. ARNI: Angiotensin receptor-neprilysin inhibitor. MRA: mineraloid receptor antagonist.

**Table 6:** Predictors of death in CHF patients.

	Alive (*n* = 21)	Dead (*n* = 22)	*p* Value	Univariate HR (95%CI)
Age (years)	76 (±15.1)	84.4 (±6.3)	0.035	1.1 (1–1.1)
LVEF ≤ 40%	1 (4.8)	7 (31.8)	0.047	1.97 (1.2–3.2)
AF or flutter	7 (33.3)	15 (68.2)	0.022	2.1 (1.1–4)
CKD	5 (23.8)	15 (59.1)	0.019	2 (1.1–3.6)
NT-proBNP *, pg/mL	747 (182–1773)	10,966 (4539–16,094)	0.003	1.065 † (1.02–1.11)
NT-proBNP * ≥ 2598 pg/mL	3 (20)	11 (91.7)	0.001	10.2 (1.5–68.5)
Hs-TnT * < 14 ng/L	5 (27.8)	0 (0)	0.052	0.48 (0.33–0.71)
NYHA ≥ III (advanced HF)	4 (19)	4 (18.1)	0.522	-
Women	10 (47.6)	12 (54.5)	0.65	-
Smoking	3 (14.3)	6 (27.2)	0.231	-
DM	8 (38.1)	13 (59.1)	0.169	-
Hypertension	19 (90.5)	19 (86.4)	1	-
Dyslipemia	11 (52.4)	16 (72.7)	0.168	-

Results are expressed as mean and (standard deviation), or number and (percentage). HR: Hazard Ratio. CI: Confidence Interval. DM: Diabetes mellitus. LVEF: Left ventricle ejection fraction. NYHA: New York Heart Association Functional Class. HF: Heart Failure. AF: Atrial fibrillation. CKD: Chronic kidney disease. NT-proBNP, N-terminal probrain natriuretic peptide. Hs-TnT: high-sensitivity troponin-T. * only for patients with biomarkers on admission. † NT-proBNP, per 1000 pg/mL.

## Data Availability

The data presented in this study are available on request from the corresponding author.

## Supplementary Materials

The following are available online at https://www.mdpi.com/2077-0383/10/2/323/s1, Table S1: Baseline characteristics between patients with and without CHF in the whole cohort before matching.
