# Supplementary material for: Prognostic Implications of Chronic Heart Failure and Utility of NT-proBNP Levels in Heart Failure Patients with SARS-CoV-2 Infection
Source: J Clin Med. 2021 Jan 17;10(2):323. doi: 10.3390/jcm10020323 (PMC7829899; doi:10.3390/jcm10020323)
Supplement: Supplementary file 1 [file jcm-10-00323-s001.pdf]

Table S1. Baseline characteristics between patients with and without CHF in the whole cohort before matching.

	Total (*n* = 872)	CHF (*n* = 43)	Non-CHF (*n* = 829)	*p*-Value
Women	386 (44.3)	22 (51.2)	364 (43.9)	0.35
Age, years	62.0 (±18.0)	80.3 (±12.1)	61.0 (±17.9)	0.001
Influenza vaccination	115 (13.2)	10 (23.3)	105 (12.7)	0.016
Smoking	208 (23.9)	9 (20.9)	199 (24)	0.540
Diabetes	173 (19.8)	21 (48.8)	152 (18.3)	0.001
Hypertension	383 (43.9)	38 (88.4)	345 (41.6)	0.001
Dyslipemia	281 (32.2)	27 (62.8)	254 (30.6)	0.001
Obesity	158 (18.1)	14 (32.6)	144 (17.4)	0.006
Ischemic cardiac disease	60 (6.9)	10 (23.3)	50 (6)	0.001
AF or flutter	70 (8)	22 (51.2)	48 (5.8)	0.001
Valvular heart disease	29 (3.3)	14 (32.6)	15 (1.8)	0.001
ACEI or ARB II	253 (29)	23 (53.3)	230 (27.7)	0.001
COPD	66 (7.6)	8 (18.6)	58 (7)	0.012
Previous Cancer	111 (12.7)	10 (23.3)	101 (12.2)	0.034
CKD	75 (8.6)	18 (41.9)	57 (6.9)	0.001
Peripheral vascular disease	34 (3.9)	7 (16.3)	27 (3.3)	0.001
Stroke	51 (5.8)	9 (20.9)	42 (5.1)	0.001
Asthma	72 (8.3)	9 (20.9)	63 (7.6)	0.005

Results are expressed as mean and (standard deviation), or number and (percentage). CHF: Chronic heart failure. ACEI: Angiotensin-converting-enzyme inhibitors, ARB II: Angiotensin II receptor blockers. COPD: chronic obstruction pulmonary disease. CKD: chronic kidney disease. AF: atrial fibrillation.
